# The Synapse as a Central Target for Neurodevelopmental Susceptibility to Pesticides

**DOI:** 10.3390/toxics4030018

**Published:** 2016-08-26

**Authors:** Aimee Vester, W. Michael Caudle

**Affiliations:** 1 Department of Environmental Health, Rollins School of Public Health, Emory University, Atlanta, GA 30322, USA; avester@emory.edu; 2 Center for Neurodegenerative Disease, School of Medicine, Emory University, Atlanta, GA 30322, USA

**Keywords:** dopamine, GABA, glutamate, neurodevelopment, neurotoxicity, organochlorine, organophosphate, pesticide, pyrethroid, synapse

## Abstract

The developmental period of the nervous system is carefully orchestrated and highly vulnerable to alterations. One crucial factor of a properly-functioning nervous system is the synapse, as synaptic signaling is critical for the formation and maturation of neural circuits. Studies show that genetic and environmental impacts can affect diverse components of synaptic function. Importantly, synaptic dysfunction is known to be associated with neurologic and psychiatric disorders, as well as more subtle cognitive, psychomotor, and sensory defects. Given the importance of the synapse in numerous domains, we wanted to delineate the effects of pesticide exposure on synaptic function. In this review, we summarize current epidemiologic and molecular studies that demonstrate organochlorine, organophosphate, and pyrethroid pesticide exposures target the developing synapse. We postulate that the synapse plays a central role in synaptic vulnerability to pesticide exposure during neurodevelopment, and the synapse is a worthy candidate for investigating more subtle effects of chronic pesticide exposure in future studies.

## 1. Neurodevelopment

Neurodevelopment is comprised of a highly orchestrated sequence of biological processes, including proliferation, migration, differentiation, and synaptogenesis, which must occur for the normal development and function of the nervous system. *In utero*, the human nervous system arises from the neuroepithelium of the neural tube around day 18. The ventricular zone contains progenitors that form all the cell types that comprise the central nervous system (CNS). These cell types include glia, which have various supportive roles such as the protection and nourishment of neurons. Neurons are the major signaling cells of the nervous system and they generally consist of a cell body with a single, outgoing axon and numerous projecting dendrites. Once neurons differentiate in the ventricular zone they then migrate to specific regions throughout the brain, using radial glia as their guides to this localization. In humans, the majority of neuronal migration is completed around month five *in utero* and neuronal axons further refine the formation of neural circuits by extending their processes along highly regulated pathways to facilitate communication with proximal or distal neurons. To guide this process, growth cones form at the end of developing axons with long, thin filopodia that respond to chemotactic signals serving to either attract or repel the advancing growth cone [1,2].

**Figure 1:**
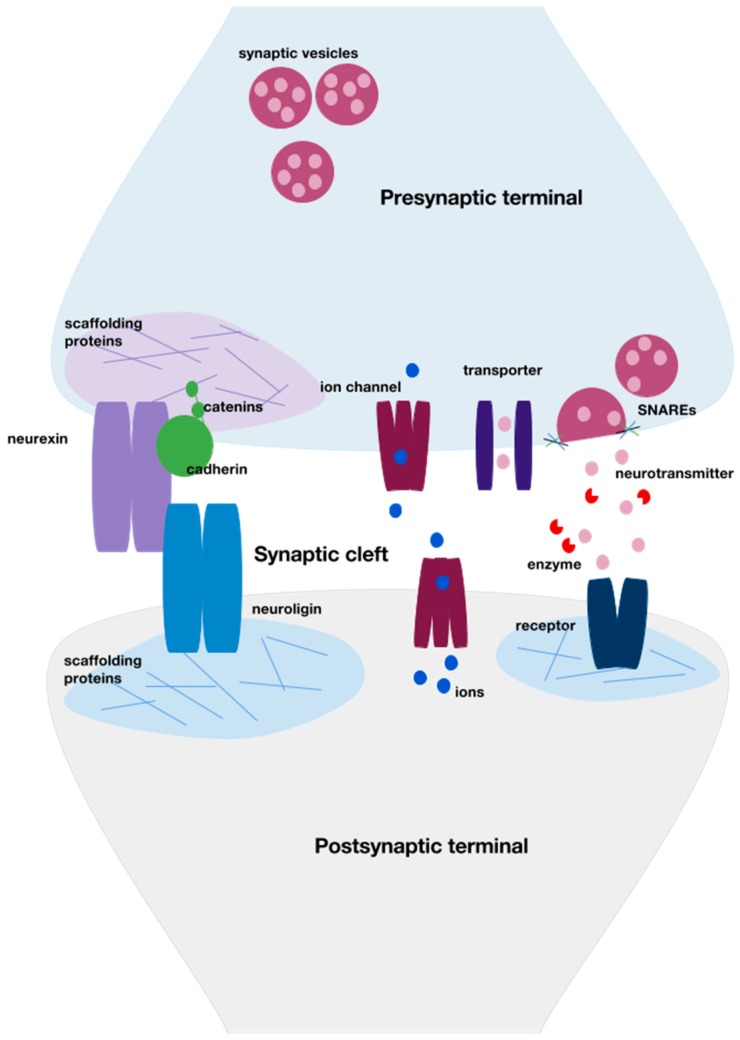
General schematic of neuronal synapse structure. A critical aspect of neurodevelopment is synaptogenesis, a highly organized series of intra- and intercellular events. These form the pre- and postsynaptic terminals and allow for neural activity between neurons. Cellular adhesion molecules, such as neurexin and neuroligin, create a physical interaction between the two terminals. The neurexin-neuroligin complex is bound on either side by scaffolding proteins, which create a specialized area within the terminal for receptors, ion channels, and transporters. Other cellular adhesion molecules such as cadherin-catenin complexes, connect actin components to the cytoskeleton scaffold and strengthen the connection. Once the synapse is formed, neurotransmitter is stored in synaptic vesicles. Some of these vesicles are attached to the presynaptic cytoskeleton as a reserve pool, awaiting depolarization via movement of ions. Once the presynaptic neuron depolarizes, the vesicle docks to the plasma membrane and fuses with it via soluble N-ethylmaleimide sensitive fusion attachment protein receptors (SNAREs). Neurotransmitter diffuses across the cleft and interacts with postsynaptic receptors. Excess neurotransmitter in the cleft is broken down via enzymes or transported back into the presynaptic terminal via reuptake transporters.

Crucial to the establishment of neural circuits are neuronal synapses, which allow for communication between neurons. When an axonal growth cone comes into contact with another neuron to form a synapse, the filopodia retract and intercellular signals from surrounding glia, the extracellular matrix (ECM) of the neuron, and other nearby neurons initiate development of the synapse. Cell adhesion is an important process for initially connecting the pre- and postsynaptic aspects of the neurons. Two important synaptic cell adhesion molecules are neurexins and neuroligins (Figure 1). The neurexins and neuroligins bind to form a trans-synaptic complex that then binds to PDZ-domain-containing proteins on either side. The PDZ domain is a protein-binding domain that binds short peptide motifs at the extreme carboxy terminal, which allows scaffolding proteins to form large modular complexes. The domain is named after the first three proteins found to contain the domain: post synaptic density protein (PSD95), Drosophila disc large tumor suppressor (Dlg1), and zonula occludens-1 protein (zo-1). The neurexin-neuroligin complex places the pre- and postsynaptic terminals in apposition and allows for further protein scaffolding construction via the two attached PDZ domains [3]. Neurexins primarily reside on the presynaptic side and induce presynaptic specialization. In contrast, neuroligins primarily reside on the postsynaptic side and initiate postsynaptic specialization. Various additional synaptic cell adhesion molecules localize to the developing synapse and are utilized to strengthen and align the connection. One such molecule is cadherin, a calcium-dependent transmembrane protein that binds with catenins, which then connect to actin components of the cytoskeleton [4] (Figure 1). Similarly, nectins also play a calcium-dependent role in cell adhesion at the developing synapse, through interactions with the actin cytoskeleton of the presynaptic active zone [2].

Following alignment of the pre- and postsynaptic terminals, the intracellular machinery necessary for the function of these elements begins to take shape. On the presynaptic side, a preassembled active zone is transported to the presynaptic terminal. This active zone is a region of the presynaptic terminal characterized by large amounts of scaffolding proteins, such as bassoon and piccolo for example, which help organize the active zone and the intracellular components necessary for normal function of the presynaptic terminal. The proteins bassoon and piccolo are large scaffolding proteins that reside at some presynaptic terminals, and they play an important role in forming the cytoskeleton structure [5]. One important function of scaffolding in the active zone is localization of neurotransmitter reuptake transporters close to the synapse. Once neurotransmitter is released presynaptically and interacts with postsynaptic receptors, these presynaptic reuptake transporters play a large role in clearing many synapses, and in regulating extracellular neurotransmitter levels. Of note, there are additional mechanisms of clearing neurotransmitter from the synapse. These mechanisms include reuptake by transporters on surrounding astrocytes, diffusion of neurotransmitter away from postsynaptic receptors, and enzymatic degradation [6].

Postsynaptically, one of the major proteins neuroligins bind to is postsynaptic density protein 95 (PSD-95), a membrane-associated guanylate kinase highly concentrated in the postsynaptic density. The postsynaptic density is a region somewhat analogous to the active zone of the presynaptic terminal. It is a densely packed region that ensures apposition of receptors to neurotransmitters diffusing across the synaptic cleft. Other scaffolding proteins that bind membrane receptors to the cytoskeleton include Shank and guanylate kinase-associated protein (GKAP) in CNS excitatory synapses [7] and gephyrin, used for γ-aminobutyric acid (GABA) receptor clustering [2,3]. Once receptors are in place, activation of these receptors via neurotransmitter released across the synapse further regulates receptor expression. Generally, increased receptor activation leads to downregulation of receptor expression and upregulation of receptor internalization [2].

Chemical synapses are polarized, allowing only unidirectional signals in a highly regulated manner. The presynaptic terminal contains thousands of neurotransmitter-filled synaptic vesicles (SVs) that are attached to the cytoskeleton as a reserve pool. To release its contents, a SV is mobilized from the cytoskeleton via phosphorylation of synapsin I and myosin II on the cytoskeleton by calcium/calmodulin kinase II (CAMKII) and myosin light chain kinase (MLCK). The SV then docks and connects to the active zone plasma membrane. Calcium (Ca^2+^) triggers membrane fusion, via soluble N-ethylmaleimide sensitive fusion protein attachment receptors (SNAREs). There are several types of SNAREs, including synaptobrevin, syntaxin 1, and SNAP-25 (synaptosomal-associated protein of 25 kDa). SNAREs form a crucial helical bundle that mediates the interaction between the vesicular and plasma membranes. Neurotransmitter left in the synaptic cleft is then taken up into the presynaptic terminal by plasma membrane transporters, while empty SVs are recycled via clathrin-mediated endocytosis [8] (Figure 1).

Once synapses are formed, their connectivity and function is continuously refined during adulthood, leading to a unique ability to form new neural networks throughout life. As part of this refinement process, heterosynaptic depression takes place. In heterosynaptic depression there is more synaptic activity in one synapse than a second related synapse, and the less active synapse regresses. Studies show that intracellular Ca^2+^ and other Ca^2+^-binding proteins are necessary for heterosynaptic depression to occur. One of these Ca^2+^-binding proteins is CAMKII, which is able to regulate the number of synaptic connections that are made and also plays a role in the regulation of cell adhesion molecules at the synapse to influence synaptic stability [2]. Thus, while the nervous system structure is largely in place early in life, refinement of neural circuits, especially at the synapse, occurs as late as the mid-twenties [9,10].

The nervous system is uniquely vulnerable to disturbances due to the need for careful organization and the fast rate of development that occurs. Synaptogenesis appears to be an especially sensitive period of neurodevelopment given its molecular complexity and elaborated length of time from gestation to adulthood [11]. Increasingly, molecular and genetic studies of neurodevelopmental diseases have pointed to alterations in synaptic proteins and their role in disease pathophysiology. For example, mutations in the cell adhesion molecules neurexins and neuroligins are associated with autism spectrum disorder (ASD) and other cognitive disorders. As discussed above, the neurexin-neuroligin complex stabilizes and connects the pre- and postsynaptic neurons, and also plays a role in synapse maturation [3]. A mouse model of an ASD-associated mutation in *Neuroligin 3* exhibits increased expression of inhibitory synaptic markers, increased frequency of inhibitory signaling, and alterations of the hippocampus and cortex [12,13]. The mice also have impaired social behavior and increased repetitive behavior, mimicking the behavioral symptoms of patients with ASD [13,14]. Similar behavioral effects are also reported in mouse models of the *Neuroligin 1* and *Neuroligin 4* genes, exemplifying that neuroligins play an important role in the synaptic pathophysiology of ASD [15,16].

Scaffolding proteins lead to the proper stabilization of synapses. Disruption of this process could potentially contribute to the observed learning and memory deficits seen when scaffolding proteins are altered. For example, detrimental changes in synaptic scaffolding and receptor transport are associated with neurobehavioral and cognitive disorders. *SNX27* is a PDZ-domain binding protein enriched in the brain, and also regulates recycling of postsynaptic receptors. Mutations in *SNX27* are associated with Down syndrome, as well as learning and memory deficits, and this is thought to be due to alterations in localization of postsynaptic α-amino-3-hydroxy-5-methyl-4-isoxazolepropionic acid (AMPA) and (*N*-methyl-D-aspartate) NMDA receptors. Mutations in *SHANK* genes are also associated with ASD, Phelan McDermid syndrome (a form of intellectual disability), schizophrenia, and Alzheimer disease (AD). Shank proteins are scaffolding proteins at glutamatergic synapses and play roles in dendritic spine morphogenesis, glutamate receptor trafficking and activity-dependent neuronal signaling [3,17]. Multiple *SHANK* isoforms have been associated with ASD, intellectual disability, and schizophrenia in genetic studies [18,19,20,21]. Mouse models lacking *SHANK2* exhibit differential ultrasound vocalization patterns, whereas mice lacking *SHANK1* exhibit altered self-grooming and marble burying behaviors that were modified by social context [22,23,24]. In mouse models carrying *SHANK3* mutations and deletions, investigators identified synaptic deficiencies in the hippocampus and striatum with concomitant increases in repetitive behaviors, social behavior deficits, impaired learning and memory, alterations in spine morphology, and glutamate receptor changes [25,26,27,28,29,30,31,32,33,34].

Beyond scaffolding, other synaptic structure proteins have also been implicated in neurodevelopmental deficits. Angelman syndrome, which leads to neurodevelopmental regression in young girls, is associated with maternal deletions of *Ube3A*. The *Ube3A* gene encodes the E3 ubiquitin ligase, which is an enzyme that marks synaptic proteins for degradation with ubiquitin. The E3 ubiquitin ligases are known to play important regulatory roles in nervous system development [35]. We know that synaptic refinement is an important component of neurodevelopment, and thus alterations in degradation processes necessary for heterogeneous depression and synaptic refinement could change the ability of stable neural circuits to form. Another important component of synapse structure is synapsins–these proteins are responsible for tethering the synaptic vesicle to the actin cytoskeleton and also assist with synaptic vesicle exocytosis. Mutations in *Synapsin* genes have been associated with behavioral and learning deficits, as well as schizophrenia. Mice lacking *Synapsin* genes have reduced object recognition and emotional memory abilities, as well as social interaction deficits [36,37]. Lastly, actin, which is a critical component of the neuronal architecture, helps form the cytoskeleton of the synapse so that scaffolding and stability can properly take place, and alterations in actin-related pathways are associated with neurodevelopmental deficits. In a knock-out mouse model of actin-depolymerizing proteins there is a deficit of actin dynamics and reduced recruitment of synaptic vesicles to the presynaptic active zone [38]. This correlates with human studies associating synaptic actin deficits with severe mental disorders [39].

In addition to synaptic structure, alterations that cause differential synaptic activity are also associated with neurodevelopmental disorders. For example, mutations in the *SNAP-25* gene have been associated with Attention-deficit Hyperactivity Disorder (ADHD) [40,41,42] and bipolar disorder [43]. *SNAP-25* is an integral part of the SNARE complex and mediates apposition of the synaptic vesicle to the presynaptic membrane for exocytosis [44]. Ablation of *SNAP-*25 in Drosophila results in a block of synaptic transmission [45,46], and in mouse models with reduced *SNAP-25* expression there are behavioral changes such as hyperactivity and impaired learning and memory [47,48,49]. Studies of Swedish children with a single nucleotide polymorphism (SNP) of the *SNAP-25* gene show decreased working memory capacity of the GG allele compared to the GA and AA alleles [44]. The SNP was also associated with gray matter density in the posterior cingulate cortex, an area previously associated with attention [50]. Studies of other SNARE proteins discovered a *de novo* mutation in the syntaxin gene *STXBP1* in a patient with Rett Syndrome, which is a disorder associated with severe cognitive impairment [51]. Mutations in the *STXBP1* gene are associated with significantly decreased neurotransmission in an in vitro model, suggesting that synaptic activity plays a role in the disease process [52]. In a mouse model, the Parkinson disease (PD)-associated mutation in *LRRK2* affects excitatory postsynaptic signaling in the hippocampus and *LRRK2* kinase activity increases as mice age, suggesting that developmental alterations of excitatory synaptic activity relate to long-term outcomes [53]. In addition to alterations in overall synaptic activity, studies have associated neurodevelopmental disease with changes in the excitatory/inhibitory balance at the synapse as well. This balance plays a role in the cortex and hippocampus in intellectual disability, ASD, schizophrenia, and AD [54], leading to differential dendritic spine morphology and maintenance [55]. Studies of schizophrenia pathophysiology have shown that hypoactivity of the NMDA receptor affects excitatory glutamate signaling, which then leads to compensatory changes in inhibitory GABA in the hippocampus, prefrontal cortex, and cingulate cortex [56].

## 2. Environmental Toxicants and Neurodevelopmental Disorders

Synapses start forming *in utero* and continue to develop and reorganize throughout life through highly coordinated cellular events. While genetics has been shown to play a critical role in these processes, recent epidemiologic and molecular work has demonstrated an important contribution of exposure to environmental chemicals on the proper development and function of the nervous system, especially at the level of the synapse [57,58,59,60,61,62,63,64,65,66,67,68].

Pesticide exposure has previously been linked to neurologic and psychiatric disease in children and adults in various epidemiologic studies. Exposure to various classes of pesticides has been associated with increased risk of ASD [59,69,70], ADHD [71,72], motor and learning impairment [73], structural brain differences [74], as well as neurodegenerative diseases, such as PD [75,76,77], and AD [78]. Pesticides may pass through the placenta based on their lipid solubility properties, and the immaturity of the blood-brain barrier early in development enables pesticide effects very early in life. The lipophilic properties of many pesticides also makes them more likely to concentrate in fatty organs such as the brain [79]. Enzymes involved in metabolizing pesticides have differential activity and expression levels throughout development, and this may also predispose the developing nervous system to elevated concentrations of toxic chemicals. For example, the paraoxonase 1 (PON1) enzyme metabolizes organophosphate compounds and plasma PON1 levels differ throughout development [80]. Additionally, developmental vulnerability to pesticide exposure can continue postnatally, as children may also be highly exposed to environmental toxicants via breast feeding. Given the wide range of neuropsychiatric disorders associated with neurodevelopmental and synaptic disruptions, as well as the multitude of molecular synaptic targets noted above, we explore the potential role of synaptic alterations caused by pesticides in the development of these disorders. Our findings are summarized for ease of reference in Table 1 at the end of this review.

### 2.1. Organochlorines

Organochlorine (OC) pesticides were mainly used from the 1930s through the 1980s but due to their long half-lives and high degree of lipophilicity persist in our environment and bodies today [123]. OCs are highly chlorinated hydrocarbons that include dichlorodiphenyltrichloroethane (DDT), dieldrin, heptachlor, and endosulfan. OCs bioaccumulate due to their lipophilicity and are known to cross the placenta and excrete in breastmilk [124,125,126,127]. Perhaps most well-known is DDT, whose manufacture and use was banned in the US in 1972 but is still in use for malaria control internationally. DDT slowly metabolizes, primarily into 1,1-dichloro-2,2-bis(p-chlorophenyl) ethylene (DDE), dichlorodiphenyldichloroethane (DDD), and dichlorodiphenylacetic acid (DDA) metabolites. The DDE metabolite is lipophilic and stores in adipose tissue, and is often used as a biomarker of exposure to DDT. While DDT has a variety of effects on neuronal targets it is most effective at holding sodium (Na^+^) channels in the axonal membrane open, which then leads to prolonged depolarization of the neuron [128,129]. Other OC compounds such as heptachlor, dieldrin, and endosulfan also inhibit the GABA_A_ receptor and Ca^2+^ ATPase, further contributing to neuronal hyperexcitation [130,131,132,133,134,135,136].

A few epidemiologic exposure studies have examined the relationship between OC pesticide exposure and neurodevelopment. These studies link prenatal OC exposure to numerous functional domains, including reduced cognitive index and memory [81,82], mental and psychomotor development, [83,84,85,137,138,139], fine motor skills [140], reflexes [86,141], social development, attentional processes, and persistently impaired visual processing [87]. Interestingly, while all offspring studied were exposed *in utero*, effects were still seen up to 11 years later. Furthermore, maternal proximity to agricultural OC pesticide use was associated with increased risk of ASD. Adult cohort studies bolster the evidence that these pesticides affect neurologic function. Although not explicitly focused on neurodevelopment, these other studies are beneficial for understanding the impact of OC exposure on neurologic dysfunction. Notably, such studies observe alterations in the same functional domains studied in children in chronically exposed adults; they perform worse on cognitive, motor, and sensory neurobehavioral tests [88,142]. Additional evidence suggests exposure to OCs is associated with neurodegeneration. Several epidemiologic studies have associated OC exposure with PD risk [75,143,144,145,146,147,148,149,150,151]. Contributing to the epidemiologic evidence are studies that identified OCs in brain samples and blood serum of patients with PD [152,153,154,155]. Additionally, OC exposure is associated with Lewy body pathology, another neurodegenerative process [89]. Taken together, these studies suggest that OCs are associated with impaired neurologic dysfunction in a wide range of functional arenas. Perhaps, common pathways and targets lead to vulnerability that appears either developmentally or later in life. Given the complexity and high level of regulation that must occur during proper neurodevelopment, there are many points at which a misstep could cause downstream consequences. Since the nervous system continues to develop throughout adulthood, these sequelae may not become clinically apparent until much later in life. Furthermore, small alterations early on could potentially cascade into more widespread neurodegenerative changes or neurodegenerative susceptibility as the brain continues to develop and refine.

Molecular and genetic studies elucidate possible pathways of neurologic insult and implicate diverse pre- and postsynaptic components. Presynaptically, our group has identified alterations in key dopaminergic proteins in response to OC pesticides in the striatum and substantia nigra [90,91,102,156]. Additionally, we have observed effects in GABAergic, glutamatergic, and dopaminergic response to endosulfan in the frontal cortex [58]. Others have identified similar dopaminergic changes upon heptachlor exposure, in addition to changes in GABA_A_, NMDA, and mGluR5 receptors in vitro [93,101,103,104]. Thus, exposure to these OC pesticides induces alterations in presynaptic transport of many neurotransmitter circuits. Altered neurotransmitter transport would likely affect synaptic activity and refinement. Postsynaptically, we observe analogous changes in GABA_A_ receptor, glutamate receptor (GluN2B), and dopamine (D2) receptor levels in the frontal cortex in response to developmental endosulfan exposure [58]. The wide range of neurotransmitter systems affected both pre- and postsynaptically might explain why neurologic effects are observed in an extensive array of functional domains. This is supported by behavioral studies showing defects in motor and cognitive areas of mice exposed to endosulfan [99], as well as tremors and hyperthermia identified in rats exposed to p, p’-DDT [157].

While there are likely changes in overall synaptic activity that occur, alterations in specific brain regions are also important to consider when examining motor, cognitive, and sensory effects. In the hippocampus, for example, developmental endosulfan exposure leads to changes in proteins that regulate the postsynaptic density, synaptic plasticity, and synaptic scaffolding (CAMKII, glutamate receptor 1 (GluR1), and tau, respectively). As one might expect, behavioral alterations also occur in the endosulfan-exposed animals, including differences in spontaneous behavior and habituation deficits [99]. Another postnatal exposure model elicited an increase in norepinephrine in the hippocampus, as well as in the brainstem [97]. Interestingly, a third study that exposed mice to endosulfan during gestation and lactation noted an increase in serotonin (5HT) in the frontal cortex, but a decrease in 5HT in the striatum [98]. Additional studies have looked at frontal cortex neurons in vitro and found a multitude of effects in response to endosulfan exposure. Mouse primary cultures of the frontal cortex have reduced synaptic puncta and neurite outgrowth, with corresponding decreased synaptogenesis in response to endosulfan exposure [58,102]. Molecular pathways associated with synaptogenesis were also altered, such as mitogen-activated protein kinase (MAPK) and phosphatidylinositol-3 kinase (PI3K)/Akt, as well as pathways associated with synaptic plasticity, such as neuronal estrogen receptors [94]. These molecular changes, as well as the associated behavioral outcomes exemplified by animal exposure models, suggest that the aforementioned epidemiologic associations may be mediated by a wide range of alterations to synaptic integrity.

Neurodegenerative studies also provide insight into some of the developmental pathways that are altered because the synapse is a target of these processes as well. Perhaps, early OC pesticide contact leads to similar neurodegenerative effects in humans over an extended period of time. Adult mice exposed to heptachlor exhibit a loss of dopamine (DA) neurons and gliosis in the substantia nigra, a pathologic hallmark of PD. Similarly, adult mice exposed to dieldrin exhibit oxidative damage in the dopaminergic system of the striatum [95]. Interestingly, OC-exposed mice have corresponding motor deficits in the pole and gait tests [93] and alterations in expression and function of the dopamine transporter (DAT) [96]. We know that loss of DA neurons in the substantia nigra is characteristic of PD, but it is possible that earlier exposure causes neurodevelopmental changes of the dopaminergic system that predispose an individual to dopaminergic circuit damage later in adulthood. This was exemplified in studies that exposed mice to dieldrin [91] and heptachlor [100] developmentally and then observed susceptibility of dopaminergic neurons to adult 1-methyl-4-phenyl-1,2,3,6-tetrahydropyridine (MPTP) exposure. Thus, alterations in dopaminergic circuitry influence synaptic activity and refinement during development, and this could potentially cause vulnerability to further dopaminergic damage later in life.

### 2.2. Organophosphates

The first commercial organophosphate (OP) pesticide, parathion, was produced in 1944. Since then, hundreds of OP pesticides have been manufactured and utilized worldwide. Mechanistically, OP function involves phosphorylation and inactivation of acetylcholinesterase. Acetylcholinesterase is the enzyme responsible for hydrolyzing the neurotransmitter acetylcholine at cholinergic synapses in the peripheral and central nervous system. Without hydrolysis, acetylcholine accumulates at the synapse, leading to over-activation of muscarinic and nicotinic receptors on the post-synaptic neuron. Sequelae include uncontrollable salivation, lacrimation, urination, diaphoresis, gastrointestinal upset, emesis, miosis, and muscle spasms due to cholinergic hyperactivity [130].

Several epidemiologic studies illustrate neurotoxic effects of OP exposure that may not be fully explained by the acute acetylcholinesterase action of OPs, as they show additional transmitter systems to also be targeted or disrupted by OP exposure. Neurodevelopmental studies have focused on cognitive, motor, and brain structure abnormalities in children exposed to OP pesticides, uncovering effects that span other neurotransmitter systems and persist well into childhood. One cohort of pregnant women in the Salinas Valley, California, looked at a more chronic exposure scenario and found the women exhibited elevated levels of OP metabolites in urine. The IQ of the offspring at seven years of age was significantly lower in the more highly exposed group [158]. Similarly, exposure to another OP pesticide—chlorpyrifos—was negatively associated with IQ and working memory index, and a Taiwanese cohort of children exposed to OPs were at an increased risk of ADHD. These studies suggest OP exposure may lead to cognitive deficits [68,72], and further cognitive effects are recapitulated in additional cohorts of children [159,160,161,162,163,164,165,166,167,168,169,170]. Studies that investigated more specific cognitive domains show deficits in attention and risk of ADHD [171,172], as well as abnormal psychomotor and reflex development [173,174,175].

Moreover, OP metabolite levels in maternal blood and urine during the third trimester are associated with decreased mental development at 12 months amongst African-Americans and Hispanics. A subset of these subjects with the *PON1* Q192R QR/RR genotype appear to have a potentiated association [141]. An additional study shows that lower maternal PON1 enzyme activity levels during pregnancy might potentiate the association between OP exposure during gestation and offspring cognitive development [60]. This is an interesting set of findings because PON1 is one of the enzymes that detoxicates OP pesticides. Differential capabilities to detoxicate OPs may lead to altered susceptibility to OP intoxication. Previous studies show that PON1 activity follows a developmental enzymatic curve, and the previously-described CHAMACOS cohort exhibits differences in PON1 activity between OP-exposed mothers and their neonates [176,177]. Thus, we now know that there is both age-related and genetic vulnerability to OP pesticides associated with PON1 expression, and that this contributes to the evidence suggesting there is neurodevelopmental susceptibility in OP exposures. Besides cognitive development, there also seem to be behavioral effects related to OP exposure. In a study of children in New York City, OP exposure during pregnancy is associated with deficits in social functioning, more so in boys and in African-Americans [178], and in another study in Shenyang, China, OP exposure during pregnancy is associated with decreased neonatal neurobehavioral development [61]. Perhaps most importantly, these aforementioned cognitive and psychomotor alterations have been shown to persist once the exposure is removed, and well into adulthood in several studies [105,179,180,181,182]. Interestingly, in addition to cognitive and behavioral deficits, OP exposure is associated with gross structural changes as well, including decreased cortical thickness and a loss of expected morphologic sex differences in the brains of offspring exposed to chlorpyrifos *in utero* [74]. Lastly, acute OP exposure in infancy, another highly vulnerable time point of exposure, is associated with deficits in verbal learning and inhibitory motor control in school-aged children [73]. Taken together, these diverse neurodevelopmental changes are probably not wholly explained by the target effects of OPs on acetylcholinesterase and instead suggest there may be a more widespread mechanism.

Contributing to the observations made in epidemiologic studies, molecular studies suggest that multiple components necessary for a properly-functioning synapse are affected in the context of OP pesticide exposure. For example, 5HT neurotransmission plays an important role in regulation and differentiation throughout the developing brain, and alterations therein would likely affect multiple functional domains. Indeed, exposure to chlorpyrifos is associated with altered expression of presynaptic 5HT transporter, postsynaptic 5HT receptors (5HT1a, 5HT2), and downstream 5HT signal transduction in various brain regions, including the forebrain and brainstem [106,107,108]. There may therefore be changes occurring in the serotonergic system during critical windows of neurodevelopment, but also during later periods of neurotransmission-related refinement of serotonergic circuits. In addition to the serotonergic system, the cholinergic system appears to be affected as well. Although AChE is the target during acute OP intoxication, chronic low-level exposure also appears to affect components of the cholinergic system both prenatally and postnatally. For instance, offspring of chlorpyrifos-exposed mice showed reductions in the presynaptic choline transporter (ChAT), which returns choline to the neuron once acetylcholine is broken down by AChE [109]. Other cholinergic system proteins, such as the vesicular acetycholine transporter (vAchT), which transports acetylcholine into synaptic vesicles for release, and muscarinic receptors, which interact with acetylcholine once it is released, are affected as well [110]. These findings correlate well with animal behavior findings that indicate offspring have learning and memory defects, and increased locomotor activity [112]. Interestingly, chlorpyrifos exposure in mice *in utero* is associated with a decrease in activity of the presynaptic ChAT, but this association occurs neonatally, disappears during weaning, and then reappears in adolescence and adulthood [111]. Given that neurodevelopment proceeds all the way through adolescence and young adulthood, it is likely that cholinergic changes affect neurotransmission across more than one developmental window. These molecular results are supported by electrophysiology studies that indicate repeated chlorpyrifos administration initially enhances and then decreases synaptic transmission in the hippocampus [183], and by behavioral studies showing that cholinergic dysfunction affects working and reference memory [112]. Further studies of postnatal OP exposure also indicate the cholinergic system is affected in ways analogous to prenatal OP exposure; there are reductions in muscarinic and nicotinic receptors that normally interact with acetylcholine, as well as reductions in transporters ChAT and vAchT [115,116,117,118,119,184,185]. These data suggest exposure in various neurodevelopmental stages can affect major neurotransmitter systems, and these effects persist, as indicated by cognitive task studies performed several months after mice were initially exposed to chlorpyrifos [113]. It has also been proposed that OP-mediated disturbances of the choline and DA systems, instead of neurotransmitter-specific system pathologies, could lead to the OP-associated neurologic outcomes [186]. Further studies indicate that changes occur not only at synapses of specific neurotransmitter systems. Rather, alterations in proteins more ubiquitously expressed in synapses occur too. For example, CAMKII plays a role in synaptic plasticity as well as learning and memory, and neonatal chlorpyrifos exposure is associated with decreased CAMKII in the hippocampus and decreased habituation in behavioral studies. A process important for learning is synaptic vesicle recycling, and synaptophysin—a CAMKII substrate—is a mediator of this process. A decrease in synaptophysin occurs in the frontal cortex of the neonatally exposed mice and this might also contribute to a common pathway that results in habituation and learning deficits [114]. Thus, various functional synapse components are altered in response to OP pesticide exposure. Perhaps the wide range of neurologic and psychiatric effects observed in epidemiologic studies are due to a common synapse pathology.

### 2.3. Pyrethroids

Exposure to pyrethroids has been characterized worldwide, and occurs via agriculture, household use, and dietary ingestion [187,188,189,190,191,192]. Pyrethroids are synthetic analogues of pyrethrins, compounds derived from *Chrysanthemum cinerariaefolium*, that bind the α subunit of voltage-gated Na^+^ channels to hold them open and cause neuronal hyperexcitability [130]. Type II pyrethroids can also inhibit GABA_A_-gated and voltage-gated chloride channels and increase excitability [193]. Off-target effects on the Ca^2+^ ATPase and voltage-gated Ca^2+^ channels have also been reported [194]. While actions at these channels do not appear to explain the acute intoxication syndromes, actions at these channels could explain some neurodevelopmental effects [195].

Of note, this is the newest class of pesticides discussed in this review. To our knowledge, few epidemiologic studies have been published and neurodevelopmental data is limited. There are a few studies, though, indicating that pyrethroid exposure is still of interest in examining adverse neurodevelopmental outcomes. Notably, children in the NHANES study with pyrethroid metabolites detectable in urine have an increased risk of ADHD [71], and this association appears to be higher in boys and with hyperactive-impulse symptoms [63]. Also, prenatal exposure to piperonyl butoxide, an additive that potentiates the action of pyrethroids, is negatively associated with cognitive and motor development at 36 months of age in a cohort of black, Dominican mothers [196]. Furthermore, there is an association between urinary pyrethroid metabolites and parent-reported behavioral problems [197] and IQ [198]. These results suggest there may be cognitive and motor sequelae associated with pyrethroid exposure. Conversely, a Thai case-control study saw no statistically significant differences in urinary pyrethroid metabolites and neurobehavioral testing at six month intervals [199]. As such, there is not yet a definitive body of work indicating pyrethroids are linked to neurodevelopmental or neurodegenerative outcomes. Taken together, however, the newer use of pyrethroids may be contributing to the lack of definitive epidemiologic data and further study of longitudinal, neurodevelopmental exposure effects are still of interest. 

Like the effects observed in OP and OC pesticide exposure, biological studies again identify synaptic components as potentially vulnerable to neurodevelopmental pyrethroid exposure. Alterations in neurotransmitter systems and synaptic structure have been illustrated. The dopaminergic system was implicated in a developmental deltamethrin exposure model, where mice exhibited increased DAT and dopamine D1 receptor expression and coincident ADHD-like behaviors, such as working memory deficits, attention deficits, hyperactivity, and impulse-like behaviors [71]. Cypermethrin exposure in the neonatal period led to alterations in GluR1 in the hippocampus and tau in the frontal cortex. As discussed earlier, GluR1 and tau both play important roles in synaptic plasticity and scaffolding, respectively. These structural changes likely contribute to the observed behavioral outcomes: decreased rearing, locomotion, and habituation in the exposed mice [99].

More of the molecular data pertains to adult models, but these studies hint at pathways likely to be affected in neurodevelopment as well. Reflecting dopaminergic changes seen in neonatal exposure, treatment of adult mice with deltamethrin and permethrin increased maximal DA uptake and permethrin treatment also increased striatal DAT levels [92,200]. This increase in DA uptake is mediated by DAT [65], and alterations in DA efflux were replicated in experiments directly injecting allethrin, deltamethrin, and cyhalothrin into the rat striatum. Perhaps, alterations in Na^+^ and Ca^2+^ channel activity in either DA synaptic terminals or GABA interneurons lead to the observed changes in DA efflux [120]. As with OC and OP pesticides, pyrethroid pesticide exposure causes changes in other neurotransmitter systems beyond DA. Administration of allethrin, cyhalothrin and deltamethrin into the rat striatum also causes changes in extracellular 5HT release. The striatum is highly innervated with serotonergic neurons, and earlier studies have shown that a reduction of 5HT leads to increased aggressive and locomotor behaviors, while a rise in 5HT leads to the opposite behavioral effects [121]. These dopaminergic and serotonergic changes could contribute to the motor and cognitive behaviors observed in epidemiologic studies and animal behavior models. Beyond neurotransmitter-specific synaptic components, pyrethroid exposure alters the balance of inhibitory and excitatory signaling. This could potentially affect neuronal signaling and synaptic activity, and the related processes of synaptic plasticity and refinement. In the hippocampus, for example, the balance of inhibitory GABAergic and excitatory glutamatergic signaling affects learning and memory. Indeed, exposure to allethrin, deltamethrin, and cyhalothrin in a rat exposure model leads to compound-specific alterations to GABA and glutamate release in the hippocampus, suggesting that these pyrethroids could play a role in cognitive outcomes observed in other studies [122]. All of these effects on synaptic function occur in adult animals, but given the high degree of coordination necessary for proper neurodevelopment, could potentially have similar or even more devastating effects in developmental models.

## 3. Summary

Considerable evidence suggests that exposure to a variety of pesticides can contribute to neurodevelopmental disorders. While the cellular and molecular underpinnings that motivate these deficits are still being investigated, it appears that the synapse is a vulnerable target for disruption by these compounds. Of the current studies, developmental exposure to OC, OP, and pyrethroid pesticides each appears to impact expression of neuronal targets critical to synaptic function. These synaptic targets include both pre- and postsynaptic components. The synaptic alterations appear to be relatively promiscuous, and not selective for a particular neurotransmitter circuit or brain region. This highlights the importance of the developing synapse for toxicant-mediated neurodevelopmental disease.

There are a multitude of neurodevelopmental effects associated with pesticide exposure, and these effects span from axonal outgrowth and synaptogenesis to synaptic activity and refinement. Given the level of complexity and organization demanded of neural circuits for proper neurologic function, it is highly likely that even subtle changes in synapse structure, function, and stability could lead to lasting modifications of cognition, movement, and sensation that are not necessarily drastic pathologically. Chronic low-level exposure of pesticides has been shown to affect various pathways related to the synapse. As such, synaptic endpoints should be considered when identifying potential harm of pesticides and other environmental exposures. Observed changes in dopaminergic, serotonergic, GABAergic, and glutamatergic neurotransmission could contribute to behavioral changes and alterations in learning and memory. In addition, changes in pathways related to synaptogenesis, such as altered expression of CAMKII, tau, and GluR1, could further contribute to the observed phenotypes.

## 4. Future Directions

Further work examining the consequences of synaptic aberrations can yield a better understanding of neurodevelopment and the progression of neurological disease in general. As exemplified in this review, the impact of altered synapse development and function is widespread and persistent. In the context of environmental toxicant exposure, the synapse appears to be a common target for damage. However, using lab-based model systems with acute, high-dose pesticide exposure may not accurately represent an environmentally-relevant exposure paradigm, which is often comprised of chronic, low dose exposures. This subthreshold exposure scenario is critical to consider as more people may be exposed in this manner, and exposed individuals likely experience unmonitored, off-target effects that could take years to manifest as neurological disorders.

Given the vast amount of pesticides currently in use and the wide range of effects synapse dysfunction has on neurologic outcomes, it is beneficial to hone in on more sensitive biomarkers of synaptic dysfunction. Developing more sensitive synaptic measures allows for a more accurate assessment of synaptic damage that occurs. Additionally, these endpoints enable detection of damage in specific neuronal populations, which then pinpoint more explicit functional outcomes. To this end, high-throughput proteomic and metabolomic techniques could be utilized to more broadly identify synapse pathology and screen multiple compounds at a faster pace. Evaluating alterations to these targets can be furthered at a molecular level by using epigenetics to pinpoint possible toxicant-induced modifications to protein expression. These protein expression modifications might then lead to an elaborated understanding of the mechanisms of synapse pathology. Previous studies show that pesticide exposure induces epigenetic changes [201] that lead to neurotoxicity. More specifically, it has been shown that exposure to the OC pesticide dieldrin increases histone acetylation and subsequently induces apoptosis in dopaminergic neurons [202]. Furthermore, exposure to the pyrethroid pesticide permethrin induces changes in DNA methylation of *Nurr1*, a transcription factor that regulates TH, DAT, and dopamine receptor expression [203]. Our group showed that exposure to the organochlorine pesticides heptachlor and dieldrin also induces changes in not only methylation of *Nurr1*, but also *Pitx3*, another transcription factor involved in regulating DAT, as well as vesicular monoamine transporter 2 (VMAT2) expression [91,100]. Thus, pesticide exposure elicits changes in DNA transcription that may precede the molecular changes discussed in this review, or that may predispose individuals to respond more significantly to continued pesticide exposures. Identifying these transcription changes will be crucial for piecing together the molecular cascades that affect synapses upon exposure to pesticides.

In developing more sensitive endpoints and advancing our understanding of neurodevelopmental effects of pesticides, it will be important to identify thresholds at which synaptic dysfunction leads to alterations in neural circuitry development and behavior. It is possible that synaptic changes that produce subthreshold developmental outcomes eventually do meet a threshold, causing neurodegeneration and other neuropsychiatric disorders. This work would contribute evidence to the fetal basis of adult disease (FOAD) hypothesis, first introduced by David Barker in the context of cardiovascular disease [204]. Barker proposed that developmental aberrations occurring in the fetal stage might in part predict adult disease outcomes. This has been studied somewhat in neurologic disease, but warrants further study—prospective animal studies show that brain inflammation associated with maternal inflammation may potentiate vulnerability to heavy metals and pesticides later in life [205], and previously-mentioned epidemiologic studies show that earlier pesticide exposure increases the risk of developing neurodegenerative diseases such as PD. Thus, adding to the currently available longitudinal studies of children exposed during neurodevelopment would provide valuable information about the potential development of neuropsychiatric disorders such as schizophrenia and bipolar disorder, which are often not seen until early adulthood, as well as the development of neurodegenerative disorders such as PD and AD, which are usually not apparent until the second half of life. Lastly, studying which brain regions are susceptible to synaptic dysfunction in response to environmental exposure enables us to anticipate important endpoints to examine in later chemical risk assessments. To do so, imaging studies could be used to analyze the entire brain in animal exposure models throughout development. Identifying regions vulnerable to specific pathologies and associated behavioral outcomes allows for more targeted exposure measurements in subsequent tissue samples. 

A large body of work has provided correlations of molecular changes and behavioral abnormalities, but there is a general lack of integration of the molecular and behavioral findings, so that there is little more beyond the associative data. Crucial to our understanding will be further functional studies that examine the mechanistic sequelae of these identified synaptic changes. This will allow the field to make causal deductions, in addition to the correlational ones, about the role of synapse effects in neurodevelopment. Functional studies might include additional electrophysiology or functional MRI studies. Discovery of new synaptic endpoints and regional vulnerability described above will contribute to the development of these functional experiments, and will allow us to prove more specific pathways. Moreover, adding epidemiologic and clinical data to molecular data will push our understanding even further. Epidemiologic studies could be used to assess genetic factors that would modify the effects of environmental exposures. For example, studying the interaction of genetic variation and pesticide exposure in neurodevelopment could provide additional information about the susceptibility of individuals to disease development. De Felice et al. recently conducted a study in which a validated idiopathic autism transgenic mouse was prenatally exposed to chlorpyrifos at a sub-toxic dose. The authors found that the chlorpyrifos-exposed mice displayed delayed motor maturation in pups, as well as an altered pattern of sexual partner interaction associated with increased ultrasound vocalization in adult males [67]. Similar studies could examine polymorphisms of synaptic protein genes and vulnerability to pesticide exposure effects. Given the extended length of neurodevelopmental processes, it would be important to study effects of continued exposure from the prenatal to adult stages. As an initial step, the neuronal synapse proves to be a valuable objective because many of the pesticide compounds currently available target synaptic components in some way. Based on the wide range of disorders linked to synaptic alterations, improper synaptogenesis and synaptic activity appears to play a central role in the development of neurologic and psychiatric disorders after pesticide exposure.

## Figures and Tables

**Table 1:** Studies associating pesticide exposure and neurologic function ^1^.

Subject	Findings	Reference(s)
Organochlorines		
Human (Children)	Decr. cognitive, quantitative, verbal, sensory, memory functions; hyporeflexia	[81,82,83,84,85,86,87]
Human (Adults)	Decr. neurobehavioral performance	[88]
	Incr. serum and brain DDE levels associated with AD	[78]
	Presence of heptachlor assoc. with Lewy body pathology	[89]
Mice	Incr. DAT, increased DA uptake, VMAT2, TH in striatum	[90,91,92]
	DA neuron loss in substantia nigra, gliosis in ventral midbrain	[93,94,95,96]
	Incr. NE in hippocampus, brainstem	[97]
	Incr. 5HT in frontal cortex, decr. 5HT in striatum	[98]
	Altered GABA_A_, GluN2B, D2 receptors in frontal cortex	[58]
	Altered CAMKII, GluR1, tau in hippocampus and frontal cortex	[99]
	Parkinsonism-like movement	[93]
	Motor, cognitive behavioral deficits	[99]
	Developmental exposure potentiates MPTP toxicity	[91,100]
Rats	Incr. DAT binding	[101]
Mouse cortex primary culture	Decr. synaptic puncta, neurite outgrowth, synaptogenesis	[56,102]
	Altered MAPK, PI3K/Akt, estrogen receptor pathways	[94]
	Incr. NMDA receptor internalization and decr. mGLUR5 levels	[103]
Mouse cerebellum primary culture	Decr. GABA_A_ and NMDA receptors	[104]
Organophosphates		
Human (Children)	Decr. IQ, working memory index, cognition	[68,72]
	Incr. risk of ADHD	[68]
Human (Adults)	Self-reported memory, fatigue, muscle strength issues	[105]
Mice	Altered 5HT transporter, 5HT receptors in forebrain, brainstem	[106,107,108]
	Reduced ChAT, vAchT	[109,110,111]
	Learning, memory defects, incr. locomotion	[112,113,114]
	Decr. CAMKII in hippocampus, synaptophysin in frontal cortex	[114]
Rats	Decr. muscarinic receptors, decr. AChE activity, ChAT, vAchT	[115,116,117,118,119]
Pyrethroids		
Mice	ADHD-like behaviors: working memory and attention deficits, hyperactivity, impulse-like behaviors	[71]
	Incr. striatal DA uptake mediated by DAT, incr. D1 receptor	[71,120]
	Altered GluR1 in hippocampus, altered tau in frontal cortex	[99]
Rats	Striatal administration causes altered extracellular 5HT release	[121]
	Incr. extracellular glutamate, decr. GABA	[122]

^1^ DDE, 1,1-dichloro-2,2-bis(p-chlorophenyl) ethylene; AD, Alzheimer Disease; DAT, dopamine transporter; DA, dopamine; VMAT2, vesicular monoamine transporter 2; GluN2B, glutamate receptor; CAMKII, calcium/calmodulin-dependent kinase II; GluR1, glutamate receptor 1; MPTP, 1-methyl-4-phenyl-1,2,3,6-tetrahydropyridine; MAPK, mitogen-activated protein kinase; NMDA, N-methyl-D-aspartate; ADHD, Attention-Deficit Hyperactivity Disorder; 5HT, serotonin; ChAT, choline acetyltransferase; vAchT, vesicular acetylcholine transporter; AChE, acetylcholinesterase; GABA, γ-aminobutyric acid.
